# Synthesis, Electrochemical and Spectroscopic Characterization of Selected Quinolinecarbaldehydes and Their Schiff Base Derivatives

**DOI:** 10.3390/molecules25092053

**Published:** 2020-04-28

**Authors:** Jakub Wantulok, Marcin Szala, Andrea Quinto, Jacek E. Nycz, Stefania Giannarelli, Romana Sokolová, Maria Książek, Joachim Kusz

**Affiliations:** 1 Institute of Chemistry, University of Silesia in Katowice, ul. Szkolna 9, PL-40007 Katowice, Poland; jakub.wantulok1@gmail.com; 2 Institute of Polymer and Dye Technology, Lodz University of Technology, Stefanowskiego 12/16, 90-924 Lodz, Poland; marcin.szala@p.lodz.pl; 3 J. Heyrovský Institute of Physical Chemistry of the Czech Academy of Sciences, Dolejškova 3, 18223 Prague, Czech Republic; andrea.quinto28@libero.it; 4 Department of Chemistry and Industrial Chemistry, University of Pisa, Via Moruzzi 13, 56124 Pisa, Italy; stefania.giannarelli@unipi.it; 5 Silesian Center for Education and Interdisciplinary Research, University of Silesia, Institute of Physics, 75 Pułku Piechoty 1a, 41-500 Chorzów, Poland; maria.ksiazek@us.edu.pl (M.K.); joachim.kusz@us.edu.pl (J.K.)

**Keywords:** vilsmeier-haack, reimer-tiemann, duff, aldehyde, aldazine, heterocyclic, cyclic voltammetry

## Abstract

A new approach to the synthesis of selected quinolinecarbaldehydes with carbonyl groups located at C5 and/or in C7 positions is presented in this paper in conjunction with spectroscopic characterization of the products. The classical Reimer-Tiemann, Vilsmeier-Haack and Duff aldehyde synthesis methods were compared due to their importance. Computational studies were carried out to explain the preferred selectivity of the presented formylation transformations. A carbene insertion reaction based on Reimer-Tiemann methodology is presented for making 7-bromo-8-hydroxyquinoline-5-carbaldehyde. Additionally, Duff and Vilsmeier-Haack reactions were used in the double formylation of quinoline derivatives and their analogues benzo[*h*]quinolin-10-ol, 8-hydroxy-2-methylquinoline-5,7-dicarbaldehyde, 8-(dimethylamino) quinoline-5,7-dicarbaldehyde and 10-hydroxybenzo[h]quinoline-7,9-dicarbaldehyde. Four Schiff base derivatives of 2,6-diisopropylbenzenamine were prepared from selected quinoline-5-carbaldehydes and quinoline-7-carbaldehyde by an efficient synthesis protocol. Their properties have been characterized by a combination of several techniques: MS, HRMS, GC-MS, FTIR, electronic absorption spectroscopy and multinuclear NMR. The electrochemical properties of 8-hydroxy-quinoline-5-carbaldehyde, 6-(dimethylamino)quinoline-5-carbaldehyde and its methylated derivative were investigated, and a strong correlation between the chemical structure and obtained reduction and oxidation potentials was found. The presence of a methyl group facilitates oxidation. In contrast, the reduction potential of methylated compounds was more negative comparing to non-methylated structure. Calculations of frontier molecular orbitals supported the finding. The structures of 8-hydroxy-2-methylquinoline-5,7-dicarbaldehyde and four Schiff bases were determined by single-crystal X-ray diffraction measurements.

## 1. Introduction

Aldehydes are a class of compounds of great interest from the synthetic, theoretical and application point of view, which can be transformed into a wide range of structural frameworks through application of a variety of reactions. Various synthetic protocols have been developed including the classical Reimer-Tiemann, Vilsmeier-Haack and Duff reactions, which are well known as versatile synthetic tools for the formylation of electron-rich aromatics. However, literature data has often inaccurately reported the position of the newly formed carbonyl group(s). Formylation of 8-hydroxyquinoline under Reimer-Tiemann condition can go to both the C5 (38%) and C7 (10%) positions [1]. On the other hand González-Vera et al. reported the formylation of 2-methylquinolin-8-ol leading exclusively to 8-hydroxy-2-methylquinoline-5-carbaldehyde in 64% yield [2] similarly to Chen et al. who formylated 8-hydroxyquinoline and obtained 8-hydroxyquinoline-5-carbaldehyde in 19.3% yield [3]. Ding. et al. only obtained 8-hydroxy-7-quinaldinecarbaldehyde, a product formylated in a different position [4]. In this study we propose the transformation of some newly formed aldehydes into sterically hindered crystalline Schiff bases.

## 2. Results and Discussion

In the current study, the formylation reactions of selected quinoline derivatives at **R**, **R^1^** and **R^2^** positions were the focus. These type compounds have not been fully exploited and may be served as interesting building blocks for synthetic purposes.

### 2.1. Synthesis and Structural Characterization

**Scheme 1:**
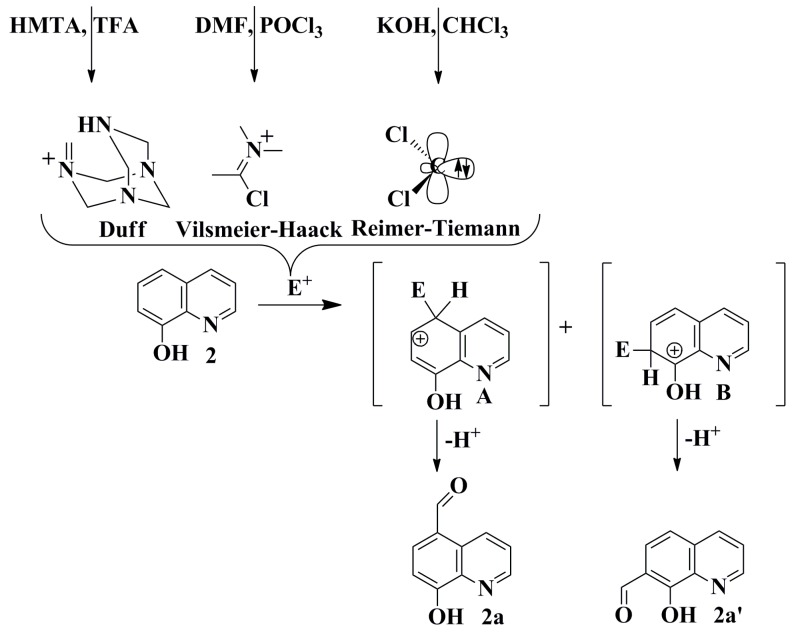
Formylation of 8-hydroxyquinoline (**1c**).

The synthesis of certain quinolinecarbaldehydes **2** were based on closely related Reimer-Tiemann (**R-T**), Vilsmeier-Haack (**V-H**) and Duff (**D**) formylation reactions (Scheme 1).

**Scheme 2:**
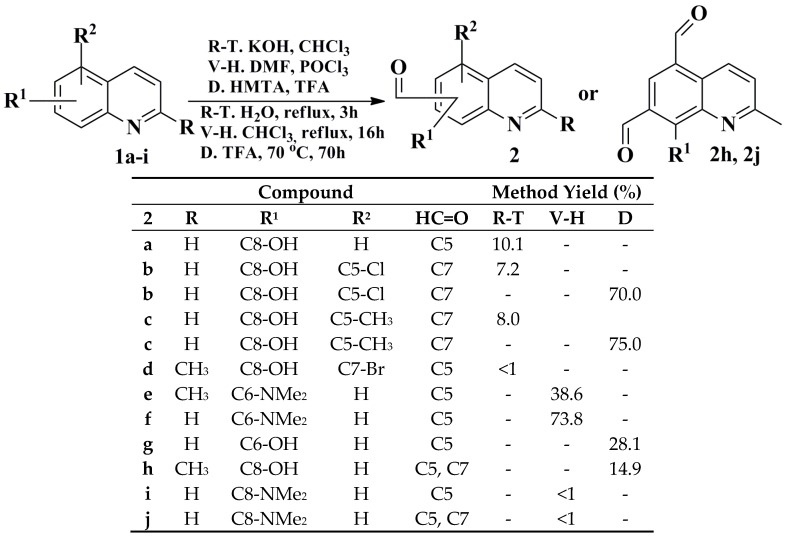
Synthesis of quinolinecarbaldehydes **2**.

**Scheme 3:**
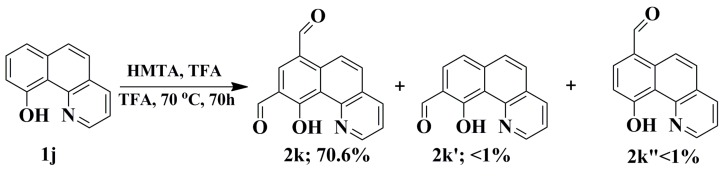
Duff type formylation of benzo[*h*]quinolin-10-ol (**1j**).

The aim of the current study is to explore selected synthetic routes, and formylated a series of derivatives of 8-hydroxyquinoline have been prepared as shown in Scheme 2 and Scheme 3.

A lone pair of electrons on the hydroxyl oxygen or dimethylamino nitrogen at **R^1^** position on phenyl ring are conjugated with the π system of quinolone, which increases the electron density at both the C5 and C7 positions on the phenyl ring in all studied quinolines [5,6]. Both positions are suitable for substitution reaction via a S_E_Ar mechanism [6]. The formulation mechanisms of Reimer-Tiemann, Vilsmeier-Haack and Duff reactions are similar to each other and to other electrophilic aromatic substitution reactions. However the initial electrophile reactions are different among these methods. In the case of the Reimer-Tiemann reaction, it is in situ Fisher type electrophilic carbene generated (in most cases dichlorocarbene), for Vilsmeier-Haack it is Vilsmeier’s reagent, and for Duff reaction it has been proposed an initial aminoalkylation followed by dehydrogenation (generated from HMTA) [7] (Scheme 1). It is important to notice that all these electophiles possess different electrophilicity. The attack by an electrophile generates a Wheland intermediate cation (or arenium ion) followed by the loss of a proton to restore the aromaticity (Scheme 1). Analogically to our recent results [6] the substitution at C5 (**R^2^**) position would have a great stabilizing effect on the adjacent carbon than at C7 (**R^1^**) position in quinoline constitution (Scheme 2). Several authors announced the similar structure of 8-hydroxyquinoline with newly formed group at C5 position on phenol ring in the same type of reactions.

**Figure 1:**
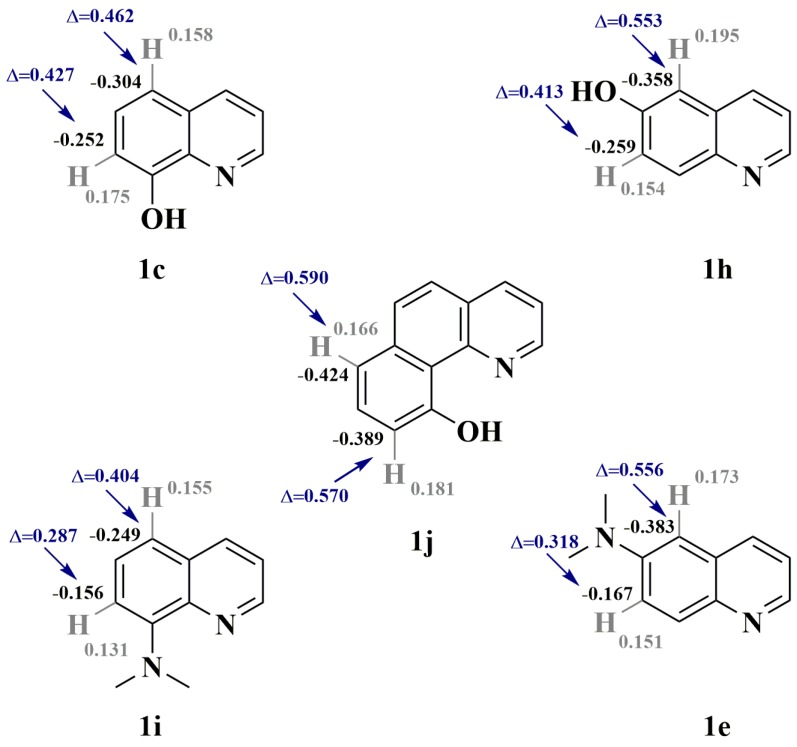
The electrostatic potentials for the selected quinolines.

The Vilsmeier’s reagent and electrophilic dichlorocarbene possess electron-withdrawing (EW) ability. One consequence of the existence of EW groups in electrophiles is a decreased electron density in the newly generated Wheland cations (Scheme 1), which deactivate the *N*,*N*-dimethylaniline (or phenol) ring for the next substitution. A dimethylamino moiety, like a hydroxyl group in the C8 position, increases the electron density at both the C5 and C7 sites, which makes double formylation leading to 8-(dimethylamino)quinoline-5,7-dicarbaldehyde (**2j**, yield < 1%) possible in the case of the Vilsmeier-Haack transformation (Figure 1). In contrast the Duff cyclic electrophile, after the generation of a Wheland cation (Scheme 1), does not deactivate the phenol ring for the next substitution, which allows the double formylation to generate 8-hydroxy-2-methylquinoline-5,7-dicarbaldehyde (**2h**) and 10-hydroxybenzo[h]quinoline-7,9-dicarbaldehyde (**2j**) in good yields. Zhang et al. have claimed an 82% isolated yield of 8-hydroxyquinoline-5,7-dicarbaldehyde in a similar procedure [8]. The absence of a methyl group in the C2 (**R**) position could have a positive influence to increase the yield of products. **R** substituents can undergo further side reactions. Additionally, in both reaction mixtures we identified monoformylated products. It is, at first sight, somewhat surprising that the formylation of 6-hydroxyquinoline (**1h**) using the Duff method led exclusively to a monoaldehyde with the newly formed carbonyl group in the C5 position (Scheme 2) [9]. The differences in reactivity between 8-hydroxyquinoline (**1c**), 6-hydroxyquinoline (**1h**), *N*,*N*-dimethylquinolin-6-amine (**1e**) and *N*,*N*-dimethylquinolin-8-amine (**1i**) could be explained by the differences in electrostatic potentials of the atoms participating in the formylation transformation (Figure 1). The formal charge of atoms in the C5-H and C7-H bonds were calculated based on electrostatic potentials, which are given in units of electrons and are shown in Figure 1. A positive charge indicates a deficiency of electrons on an atom and a negative charge, an excess of electrons. The electrostatic potential for a hydrogen atom is marked in gray (positive value) and for carbon in black (negative value). The differences in their potential are marked in blue. The higher difference in atomic charges between C5 and C7 positions show the preference of selected monoformylations products with novel carbonyl group only in C5 position. The difference of electrostatic potential of C5-H bond 0.556 for **1e** suggests the easier cleavage of H atom from C5, compared to C7-H with electrostatic potential difference 0.318 (for **1h**, similarly). The smaller differences in the atomic charges difference of atoms in bonds C5-H and C7-H suggest the possibility of double formylation and the presence of both regioisomers with new carbonyl group in C5 and C7 positions (Figure 1).

The two transition states presented in Scheme 1 would be similar in terms of structure between intermediate **A** and **B**. If we accept Scheme 1 as a model for the formylation, a substitution at the C5 position would have a greater stabilizing effect on the adjacent carbon than that at the C7 position in the quinoline scaffold. This discrepancy between the electron density and preferred position of substitution was explained by Olah, as the weaker electrophiles (or with less nucleophilicity of aromatics) showed higher substrate selectivity. The transition states are of a “late” nature resembling the intermediates and the *ortho*/*para* ratio decreases, with *para*-substitution becoming predominant [10]. Formylation of 2-methylquinolin-8-ol (**1b**) and 8-hydroxyquinoline (**1c**) in all the presented methodologies led to complicated reaction mixtures. However, it was noticed that dialdehydes **2h** and **2k** were easy to isolate because of their low solubility in most solvents. In this case isolation relies on filtration, followed by washing with chloroform and methanol. In the reaction mixtures of the Reimer-Tiemann and Vilsmeier-Haack transformations the presence of two possible regioisomers with newly formed carbonyl groups at the C5 and C7 positions was detected by ^1^H-NMR and GC-MS techniques for the first time (Scheme 1 and Scheme 2, Figure S1e in the Supplementary Data). For the Reimer-Tiemann reaction both regioisomers were obtained in a 35:28 ratio (**2a**:**2a’**) (Figure S1e). These regioisomers with tr_C5-C=O_ = 6.024 min. and tr_C7-C=O_ = 6.360 min. possess similar mass spectral patterns, and the same *m*/*z* (Figure S1c,d, Supplementary Data) and display characteristic *m*/*z* = 173 M^+^ molecular ions, and *m*/*z* = 144 and 116 fragment ions. All the presented quinolinecarbaldehydes **2** show a preferential fragmentation with the initial loss of a carbonyl group, followed by the loss of C=O of the phenolic ring and further decomposition (Supplementary Data).

The ^1^H-NMR spectra of molecules **2** showed distinctive H-1 signals from the HC=O proton of carbonyl group located at the C5 and C7 positions (or C7 and C9 in the case of heterocycle **2k**) with chemical shifts of ca. 10.1 and 10.5 ppm, respectively. The analysis of the trends in ^1^H-NMR chemical shifts revealed that the presence of intramolecular hydrogen bonds between neighboring carbonyl group located at C7 position and hydroxyl or dimethylamino group at C8 position increased the deshielding effect, resulting in the low-field signals. The chemical shifts in DMSO solution were moved to downfield (larger δ; 10.5 ppm), while the higher field signal (10.1 ppm) is for a carbonyl group located at C5 position, where intramolecular hydrogen bonds were absent. In contrast, the proton of HC=N group for molecules **3** (Scheme 5) are located at C5 (8.51 ppm) and C7 (8.38 ppm). The ^13^C-NMR spectra showed an opposite effect to the ^1^H-NMR ones. The carbon atoms of the carbonyl groups located at the C5 and C7 positions showed distinctive signals with ^13^C chemical shifts of ca. 192 and 188 ppm, respectively. The chemical shifts were moved to downfield (larger δ) for a non-protonated carbonyl group at the C5 position with resonance signals at ca. 192 ppm. The deprotonation of the carbonyl group makes the carbon atom more positive, which moves the chemical shift downfield (larger δ). In the constitution of molecules **3** (Scheme 5), the carbon atoms of the HC=N group located at C5 (163 ppm) and C7 (165 ppm) positions showed opposite effects, which can be explained in the frame of electron density.

**Figure 2:**
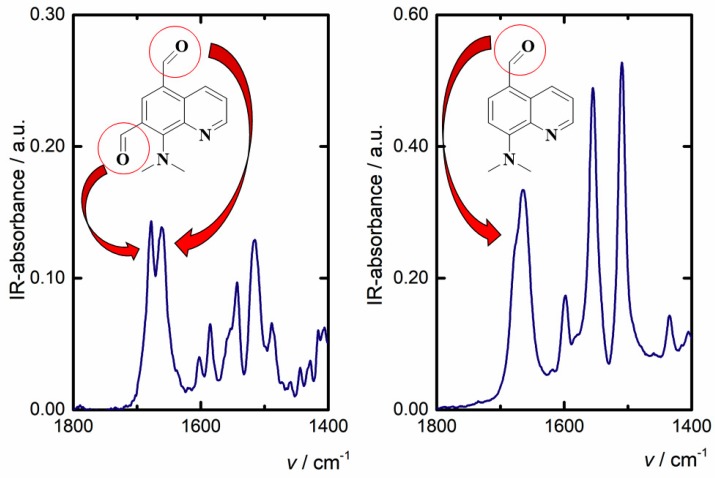
IR spectra of **2j** (**left**) and **2i** (**right**).

The IR spectra of molecules **2** showed distinctive carbonyl signals for the groups located at C5 and C7 [or C7 and C9 in the case of heterocycle **2k**] positions in the range 1663–1686 νC=O. The analysis of the trends suggests that carbonyl group located at C5 possess rather smaller values than that at C7. In the case of **2j**, for the first time we were able to detect two signals from both carbonyl groups located at C5 and C7 (Figure 2).

Comparison of the double formylated products **2h** and **2j** revealed that the presence of intramolecular and intermolecular hydrogen bonds between the carbonyls and hydroxyl groups has the impact on overlapping signals (Figure 2). This could explain why compound **2j** possesses a dimethylamino group at the C8 position instead of a hydroxyl group and has two separate signals at 1678 ν_C=O_ and 1661 ν_C=O_.

Comparing the yields of heterocycles **2** presented in Scheme 2, the Duff methodology appeared superior to the Reimer-Tiemann and Vilsmeier-Haack reactions for formylation of phenol derivatives, and gave a lower yield for *N,N*-dimethylaniline derivatives. More attention has been paid to the Reimer-Tiemann reaction and the generation of Fisher type electrophilic carbenes to show its potentially rich chemistry. One of the characteristic reactions of the carbene moiety is insertion reactions. DeAngelis et al. reacted an initially generated dichlorocarbene with indoles to form a ring expansion or a dichloromethyl-substituted product [11]. We applied the Reimer-Tiemann methodology the first time to show carbene insertion into a C-Br bond to produce 7-bromo-8-hydroxyquinoline-5-carbaldehyde (**2d**). Under standard Reimer-Tiemann conditions, molecule **1a** and other reagents were irradiated by a 75 W lamp to initiate the reactions in the presence of carbenes. Although relatively higher yields was observed during irradiation, unfortunately, the yield of this preliminary reaction is not satisfactory yet (Scheme 1).

**Scheme 4:**
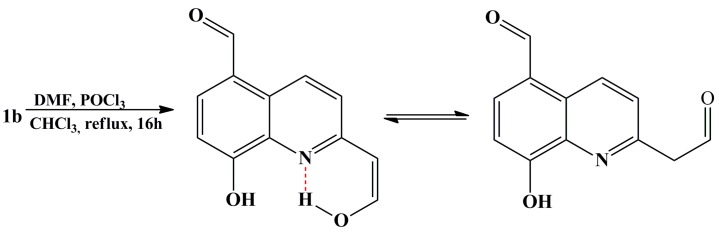
Synthesis of (*Z*)-8-hydroxy-2-(2-hydroxyvinyl)quinoline-5-carbaldehyde (**2l**). Intramolecular hydrogen-bonding interactions influencing the conformation of molecule **2l**.

The Reimer-Tiemann and Vilsmeier-Haack reactions occur at a basic environment in contrast to the Duff protocol which occurs in an acidic medium. A negative impact on the synthesis of quinolinecarbaldehydes **2** was noticed during final stage when a basic environment was applied in hydrolysis reactions. This is not surprising, because aromatic aldehydes can disproportionate in strongly basic solutions according to well-known Cannizzaro reaction mechanism, especially during long time exposure. To avoid this possible reactivity in a basic environment the reaction time was reduced to three hours for the Reimer-Tiemann protocol. The next limitation or potentially a further development for both Reimer-Tiemann and Vilsmeier-Haack reactivity could be linked to the presence of a methyl group at the **R** position in the quinoline skeleton. The newly formed aldehydes can be reacted under base-catalyzed condensation reactions, such as the Perkin transformation, leading to styryl type compounds. Like Nandhakumar et al. we isolated the product from the reaction between the electrophilic Vilsmeier’s reagent generated in situ and the methyl group located at the **R** position in the quinoline ring [12]. After hydrolysis a moderate yield of (*Z*)-8-hydroxy-2-(2-hydroxyvinyl)quinoline-5-carbaldehyde (**2l**) was isolated (Scheme 4). This product adopts a *Z* conformation due to the presence of a pseudo-ring which is stabilized by intramolecular hydrogen bonding (Scheme 4). The small *J*_H,H_ coupling constants proved the presence of *Z* conformer over *E*, due to the Karplus equation. The molecule **2l** is similarly to other quinolinecarbaldehydes **2** which had preferential fragmentation with the initial loss of carbonyl group (Figure S6d).

As presented above, the reactivity between the electrophilic Vilsmeier’s reagent and the methyl group located at the C2 position in the quinoline moiety could be explained by the electron density at the C2 (−0.714 **1b**) position. It is important to pay attention to the hazards of the Vilsmeier-Haack reaction due to its thermal instability of the Vilsmeier intermediate, especially for multigram scale reactions [13]. A further limitation of the Vilsmeier-Haack reaction was described by Morimura et al. Derivatives of 8-hydroxyquinoline like other phenol-type reagents possess hydroxyl nucleophilic substituents that can react with POCl_3_ and the Vilsmeier’s reagent leading to aryl formates [14].

The Duff reaction occurring in acidic environments also has some limitations. The low yield obtained in the formylation of amine derivatives is a result of the acidic environment used during the procedure. The nitrogen atoms of molecule **1e** are protonated in an acidic environment and consequently form a dimethylaminium group in situ at the C6 position which does not activate the benzene ring to facilitate aromatic electrophilic substitution, and consequently does not undergo the Duff reaction. In this case the starting material was recovered (Scheme 1).

**Scheme 5:**
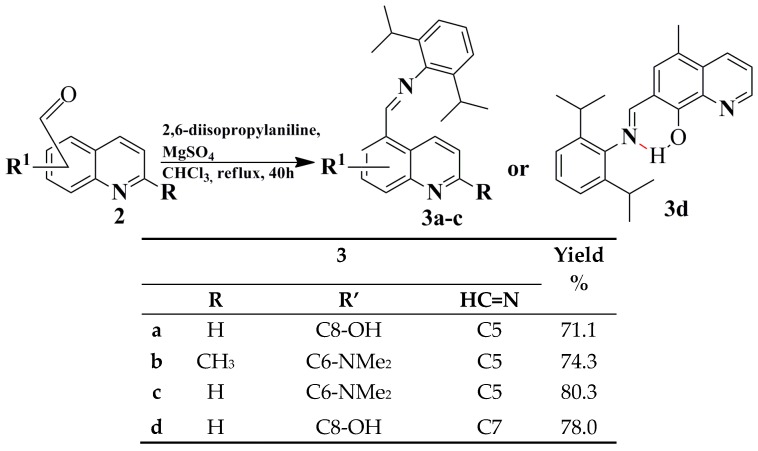
Synthesis of Schiff bases **3**. R = H, CH_3_, R’ = Me, C8-OH, C6-NMe_2_.

Some selected quinolinecarbaldehydes **2** were reacted with 2,6-diisopropylbenzenamine as an example of a primary amine giving four crystalline Schiff bases with yields up to 80% (Scheme 5). The addition of a 2,6-diisopropylbenzenamine nucleophile to heterocycles **2a**, **2c**, **2e** or **2f** is a nucleophilic addition–elimination reaction. The final products are an imine-Schiff base and water. As a reversible reaction, in acidic aqueous solutions, the products are hydrolyzed back to the aldehydes and amine. The equilibrium favors the nitrogen-protonated tetrahedral intermediate because nitrogen is more basic than oxygen. The equilibrium can be forced toward the imine species by removing water as it is formed. MgSO_4_ was used as dehydrating agent. The choice of 2,6-diisopropylbenzenamine is because it easily forms high quality crystals [15].

### 2.2. Crystal Structure Determination and Refinement

Data for the molecules **2h**, **3a**, **3b** and **3d** were collected on a SuperNova diffractometer using an Atlas CCD detector, while the measurement of compound **3c** was performed on an Xcalibur diffractometer with a Sapphire 3 CCD detector. Graphite-monochromated Mo-K_α_ radiation was used for all measurements. The crystals of compounds **2h**, **3a**, **3b** and **3d** were cooled down by a cold dry nitrogen gas stream (Oxford Cryosystems equipment), while the crystal of compound **3c** was measured at room temperature. The temperature stability was ±1 K. The structures were solved by direct methods using SHELXS-2013 program and refined by full-matrix least-squares on F^2^ (all data) using the SHELXL-2014/7 program [16]. All non-hydrogen atoms were refined anisotropically. All H atoms bound to C atoms were refined using a riding model with C–H distances of 0.95 Å (aromatic), 0.98 Å (methyl) or 1Å (methine) and Uiso(H) values of 1.2 Ueq(C) or 1.5 Ueq(C). Hydroxyl H atoms were refined with distances of 0.84 Å and Uiso(H) values of 1.5 Ueq(O). Details concerning crystal data and refinement are gathered in Table 1 and Table 2.

### 2.3. X-ray Studies

**Figure 3:**
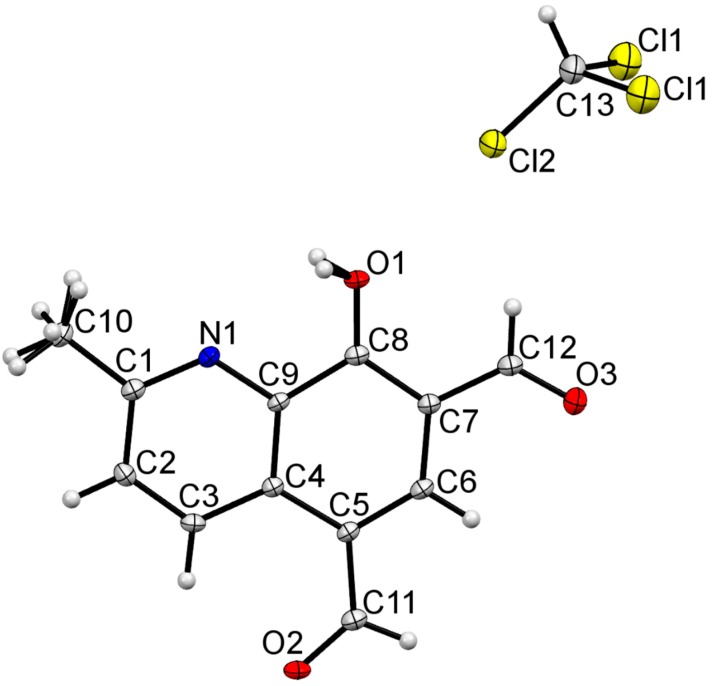
ORTEPs drawing of compound **2h** with 50% probability displacement ellipsoids.

**Figure 4:**
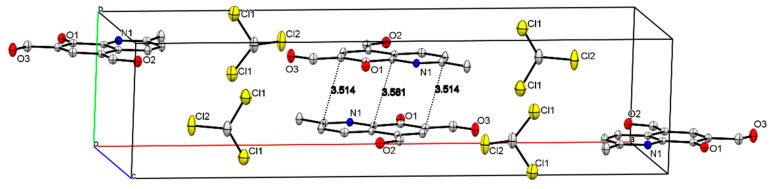
The π-stacking interactions in cell packing of compound **2h**.

8-Hydroxy-2-methylquinoline-5,7-dicarbaldehyde (**2h**) crystallizes with chloroform in the Pnma space group (Figure 3). The molecular ring systems are both essentially planar. The packing of the compound **2h** in the structure is stabilised by parallelly-displaced π-π stacking interactions, forming a π-stacking interaction as illustrated in Figure 4. The centroid–centroid distances is 3.544 Å and the shift distances is 1.451 Å. Compound co-crystallised with chloroform is associated through strong C8-O1H···Cl2C13 hydrogen bonds. In the crystal structure of compound **2h** several intra- and intermolecular hydrogen bonds are observed. To the best of our knowledge, this is first time that the X-ray structure of quinolinecarbaldehyde has been reported.

**Figure 5:**
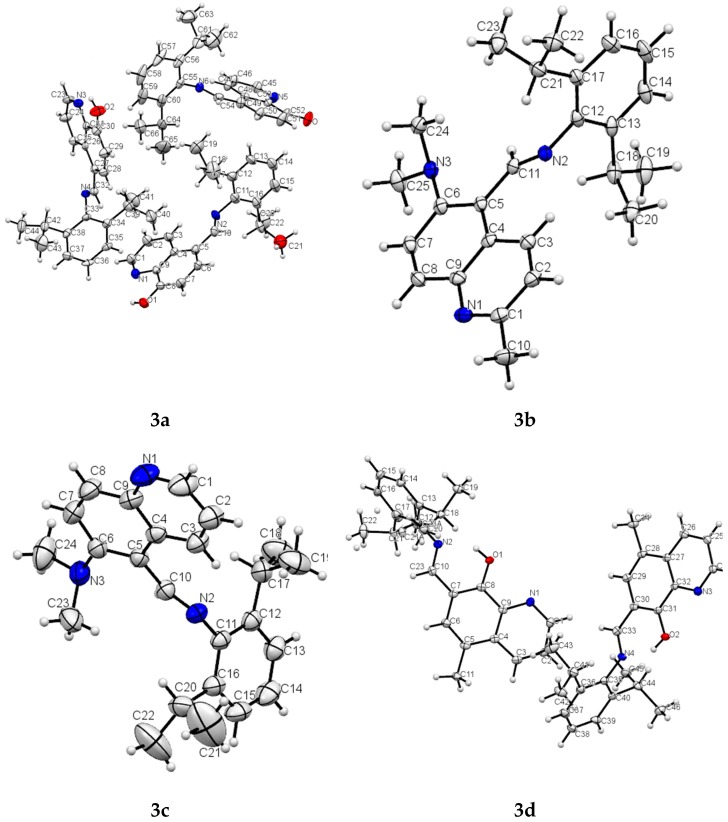
ORTEPs drawing of Schiff bases (**3a**), (**3b**), (**3c**) and (**3d**) with 50% probability displacement ellipsoids.

The molecules **3a**, **3b**, **3c** and **3d** were crystallized in the trigonal, orthorhombic, monoclinic and monoclinic space groups, respectively, which are displayed as ORTEP representations in Figure 5. Details concerning crystal data and refinement are gathered in Table 1 and Table 2.

The phenyl and quinoline rings in the compounds adopt an *E* configuration along the imino functional group. In the Schiff base compounds both the planes of the quinoline and the phenyl moieties are aligned almost perpendicularly at angles of 89.64° (**3a**), 87.51° (**3b**), 78.65° (**3c**) and 79.98° (**3d**), respectively. The –C=N– bond lengths of 1.27 Å are typical *E* Schiff base compounds **3a**, **3b** and **3c**, the –C=N– bond length of 1.28 Å for compound **3d** is longer due to participation in pseudo-ring. The substituents located on the phenol ring in molecule **3d** and on the imino group show their ability to form a pseudo-ring, which is stabilized by intramolecular hydrogen bond indicated on Scheme 5. The consequence of the formation of pseudo-ring in compound **3d** constitution is the presence of strongest hydrogen bond (O1–H1−N2) among all presented Schiff bases **3**. The structures of the presented compounds are stabilized by hydrogen bonds.

### 2.4. Electrochemical Measurements

**Figure 6:**
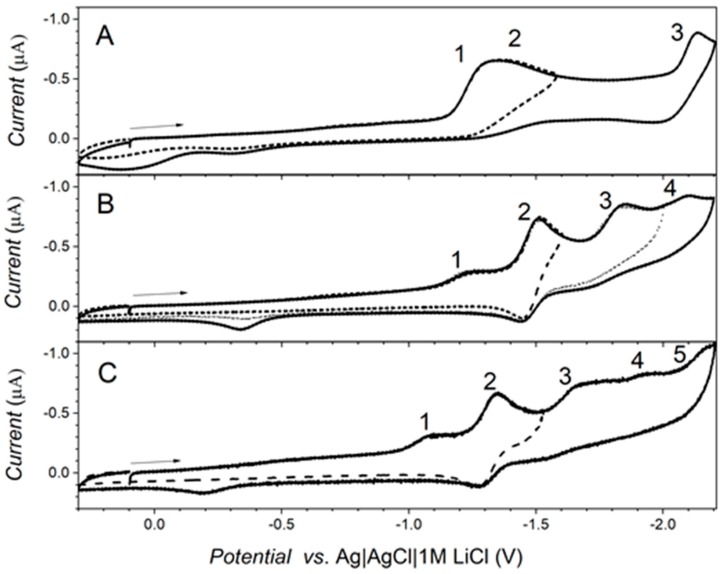
Cyclic voltammograms of 0.2 mM **2a** (**A**), **2e** (**B**) and **2f** (**C**) in acetonitrile and 0.1 M TBAPF_6_ on glassy carbon electrode.

Electrochemical measurements were performed on a glassy carbon electrode in acetonitrile. Compound **2a** yields three reduction waves in a range of potentials from 0.1 V to −2.2 V (Figure 6A). The first reduction wave at −1.300 V is followed by reduction wave 2 at a peak potential −1.400 V. The charge consumed during the exhaustive electrolysis behind the second reduction wave corresponded to one electron.

**Figure 7:**
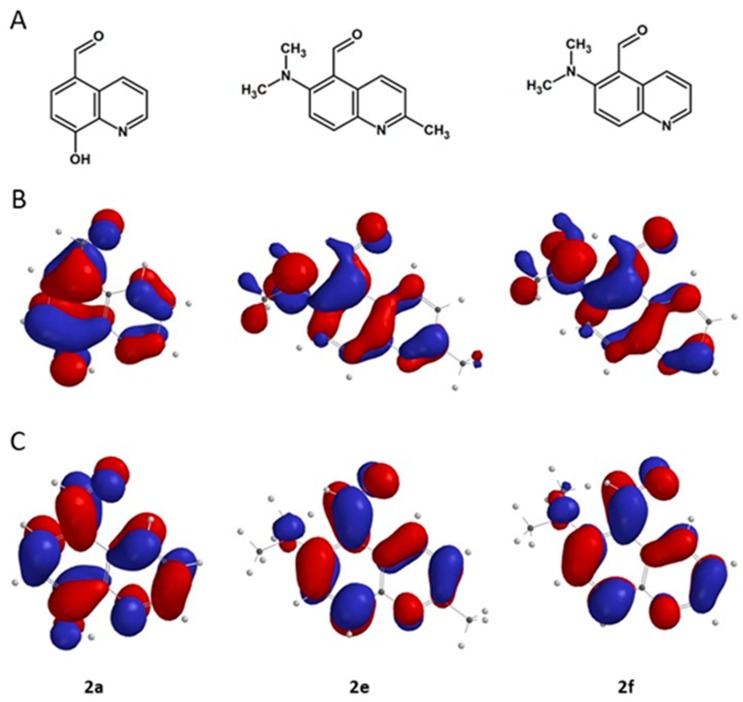
Chemical structures of compounds **2a**, **2e** and **2f** (**A**), the spatial distribution of the HOMO orbitals (**B**) and LUMO orbitals (**C**).

The third one-electron reduction wave occurred at −2.133 V, the potentiostatic electrolysis at −2.1 V resulted in the consumed charge corresponding to two electrons. Such a discrepancy in number of electrons suggests that the first two reduction waves correspond to different dissociation forms of compound **2a**, which are present in solution under experimental conditions; the consumed charge corresponds to the sum of their concentrations. The spatial distribution of LUMO orbitals suggests that carboxyl group accepts the first electron (Figure 7C). The reduction of aldehyde is usually two-electron and two-proton process according to literature [17] We suggest that hydroxyl group present in the chemical structure of compound **2a** (Figure 7A) can serve as a proton donor, which participate in reduction process. Such effects were found in literature in the case of reduction of hydroxylated benzonitriles, where half molecules present in solution served as proton donors and the charge after the exhaustive electrolysis corresponded to the reduction of half molecules present in solution [18,19]. The study of this effect is not the aim of this manuscript and will be investigated in further study.

Cyclic voltammogram obtained for compound **2e** yields four reduction waves up to −2.2 V (Figure 6B). All reduction waves increase linearly with concentration. The first reduction wave at −1.233 V is irreversible, the second one-electron wave at −1.510 V is quasi-reversible. The peak-to-peak separation of the wave **2** is |*E*_p_^c^ − *E*_p_^a^| = 68 mV. This behavior is similar to that of compound **2f**, which yields five reduction waves in the same range of potentials. The first irreversible reduction wave of **2f** occurs at −1.087 V and the second reversible one-electron reduction wave with peak-to-peak separation of |*E*_p_^c^ − *E*_p_^a^| = 59 mV occurs at −1.346 V. Most likely, a radical anion is formed at the first reduction wave, it is delocalized over the whole π-conjugated molecule as shown by LUMO spatial distribution (Figure 7C) and a fast protonation follows. The formed radical can undergo a dimerization process or is further reduced to form corresponding alcohol. The latter can be preferential under used experimental conditions, because the exhaustive electrolysis at the potential behind the second reduction wave resulted in charge consuming corresponding to two electron process. We tend to investigate the reduction mechanism in details in further study. Importantly, the peak potential of the first reduction peak of compound **2e** occurs at more negative potential than Ep^1^ of compound **2f**. The difference is caused by induction effect of methyl group. This agrees with calculated energies of LUMO orbitals for both compounds, which increase in order *E*_LUMO_(**2e**) = −1.89 eV < *E*_LUMO_(**2f**) = −1.97 eV.

Oxidation properties were studied by means of cyclic voltammetry (Figure S1, Supplementary material). Cyclic voltammetry of **2a** yields two oxidation waves at potentials 1.349 V and 1.637 V. The charge consumed during the exhaustive electrolysis of compound **2a** at the potential behind the first oxidation wave corresponded to two electrons. According to Figure 7B, the electroactive site for oxidation is most likely hydroxyl group in para position to the aldehyde. Compound **2e** yields better defined oxidation waves at *E*_1_ = 1.276 V and *E*_2_ = 1.662 V and oxidation waves at E_1_ = 1.385 V and *E*_2_ = 1.765 V were registered for compound **2f**. According to the spatial distribution of HOMO orbitals (Figure 7B) the amine can be oxidized. According to the literature, oxidation can yield a dealkylated product [20], and tail to tail coupling is also known in the literature for dialkylanilines [17]. The energies of HOMO orbitals calculated for the molecule in a vacuum suggest that compound **2e** is more easily oxidized than compound **2f**; *E*_HOMO_(**2e**) = −5.67 eV > *E*_HOMO_(**2f**) = −5.77 eV, which is in agreement with the experimental results.

## 3. Materials and Methods

### 3.1. Materials

All experiments were carried out in an atmosphere of dry argon and flasks were flame dried. Solvents were dried by usual methods (diethyl ether and THF over benzophenone ketyl, CHCl_3_ and CH_2_Cl_2_ over P_4_O_10_, hexane over sodium-potassium alloy and DMF over molecular sieves) and distilled. Chromatography was carried out on Silica Gel 60 (0.15–0.3 mm, Macherey-Nagel GmbH & Co. KG, Düren, Germany). 2,2,2-Trifluoroacetic acid (Caution! TFA is a known nigrostriatal neurotoxin, and therefore compounds of this class should be handled using disposable gloves in a properly ventilated hood), 5,7-dibromo-2-methylquinolin-8-ol (**1a**), hexamethylenetetramine (HMTA), 2,6-diisopropylbenzenamine, 2-methylquinolin-8-ol (**1b**), 8-hydroxyquinoline (**1c**) and benzo[*h*]quinolin-10-ol (**1j**) were purchased from Sigma–Aldrich (Poznan, Poland), and were used without further purification. 5-Chloroquinolin-8-ol (**1d**), *N*,*N*-dimethylquinolin-6-amine (**1e**), 5-methylquinolin-8-ol (**1f**), *N*,*N*,2-trimethylquinolin-6-amine (**1g**), quinolin-6-ol (**1h**) and *N*,*N*-dimethylquinolin-8-amine (**1i**) were synthesized according to our procedures described in the literature [21,22,23,24].

### 3.2. Instrumentation

NMR spectra were obtained with Avance 400, 500 and 600 spectrometers (Bruker, Billerica, MA, USA) operating at 600.2, 500.2 or 400.1 MHz (^1^H) and 150, 125.78 or 100.5 MHz (^13^C) at 21 °C. Chemical shifts referenced to ext. TMS (^1^H, ^13^C) or using the residual CHCl_3_ signal (δ_H_ 7.26 ppm) and CDCl_3_ (δ_C_ 77.1 ppm) as internal references for ^1^H and ^13^C-NMR, respectively. Coupling constants are given in Hz. For GC-MS a 7890A gas chromatograph (Agilent Technologies, Wilmington, DE, USA) equipped with a MS (70 eV) 5975 EI/CI MSD, and a 7693 autosampler with an Agilent HP-5MS capillary column (30 m × 250 μm × 0.25 μm)—press. 127.5 kPa, total flow 19 mL/min, col. flow 2 mL/min, split—7:1, temp. prog. (70 °C—hold 0.5 min, 70–290 °C/25 °C/min, 290 °C—hold 6 min) was used. The LCMS-IT-TOF analysis was performed on an Agilent 1200 Series binary LC system coupled to a micrOTOF-Q system mass spectrometer (Bruker Daltonics, Bremen, Germany). High-resolution mass spectrometry (HRMS) measurements were performed using a Synapt G2-Si mass spectrometer (Waters, New Castle, DE, USA) equipped with an ESI source and quadrupole-time-of-flight mass analyser. To ensure accurate mass measurements, data were collected in centroid mode and mass was corrected during acquisition using leucine enkephalin solution as an external reference (Lock-Spray^TM^). The results of the measurements were processed using the MassLynx 4.1 software (Waters, Milford, CT, USA) incorporated within the instrument. A iS50 FTIR spectrometer (Nicolet, Waltham, MA, USA) was used for recording spectra in the IR range 4000–400 cm^−1^. FTIR spectra were recorded on a Perkin Elmer (Schwerzenbach, Switzerland) spectrophotometer in the spectral range 4000–450 cm^−1^ with the samples in the form of KBr pellets. Elementary analysis was performed using Vario EL III apparatus (Elementar, Langenselbold, Germany). Melting points were determined on MPA100 OptiMelt melting point apparatus (Stanford Research Systems, Sunnyvale, CA USA) and are uncorrected.

### 3.3. Electrochemical Measurements

Electrochemical measurements were carried out in 0.1 M TBAPF_6_ in acetonitrile. Cyclic voltammetry as well as exhaustive electrolysis were performed using a PGSTAT 12 AUTOLAB potentiostat (Metrohm Autolab, Utrecht, The Netherlands). A glassy carbon electrode (diameter 1 mm), a platinum net and an Ag|AgCl|1M LiCl electrode were used as the working, the auxiliary and reference electrode, respectively. Oxygen was removed from the solution by passing a stream of argon (99.998%, Messer, Graz, Austria).

### 3.4. Theoretical Calculations

Calculations of molecular orbital energies were performed using the density functional theory (DFT) calculations employing the B3LYP functional and 6-31G* basis set with Spartan ’14, v.1.1.8 software (Wavefunction, Inc. Irvine, CA, USA).

### 3.5. General Procedures for Synthesis of Selected Quinolinecarbaldehydes Based on the Reimer-Tiemann Protocol

Potassium hydroxide (14.0 g; 250.0 mmol) in water (15 mL) was added into the solution of **1c**, **1d** or **1f** (34.5 mmol) in ethanol (20 mL) and the resulting reaction mixture was brought to a gentle reflux. Chloroform (8.3 mL; 103.5 mmol) was then added dropwise to the reaction mixture over the course of 1 h. The resulting red mixture was refluxed for another 3 h, and then was cool down to room temperature. The obtained suspension was acidified by an aqueous solution of hydrochloric acid (1%) to pH ca. 7, and then volatiles were evaporated under reduced pressure. The resulting solid was dried over P_4_O_10_ and extracted at Soxhlet apparatus (chloroform). From the resulting solution the volatiles were evaporated under reduced pressure, and finally the crude product was purified on a silica gel chromatography with CH_3_Cl/MeOH (3:1) as eluent, and purified by crystallization from CH_3_Cl/hexane to yield precipitates as follows:

*8-Hydroxyquinoline-5-carbaldehyde* (**2a**) beige 0.6 g (3.5 mmol, 10.1%) [25]; m.p. = 171.3–171.8 °C; ^1^H-NMR (DMSO–*d*_6_; 400.2 MHz) *δ* = 7.26 (d, ^3^*J*_H,H_ = 8.0 Hz, 1H, aromatic), 7.78 (dd, ^3^*J*_H,H_ = 8.6 Hz, ^4^*J*_H,H_ = 4.1 Hz, 1H, aromatic), 8.17 (d, ^3^*J*_H,H_ = 8.1 Hz, 1H, aromatic), 8.97 (dd, ^3^*J*_H,H_ = 4.1 Hz, ^4^*J*_H,H_ = 1.6 Hz, 1H, aromatic), 9.56 (dd, ^3^*J*_H,H_ = 8.6 Hz, ^4^*J*_H,H_ = 1.6 Hz, 1H, aromatic), 10.14 (s, 1H, HC=O); ^13^C{^1^H}-NMR (DMSO–*d*_6_; 100.6 MHz) *δ* = 110.8, 122.4, 124.6, 126.8, 133.0, 138.0, 140.2, 149.0, 159.6, 192.2; GC-MS: t_r_ = 6.024 min, (EI) *m*/*z* (rel. int.) M^+^ = 173 (100%); (M − HCO)^+^ = 144 (17%); UV-Vis (methanol; λ [nm] (logε)): 395 (3.04), 322 (3.89), 263 (3.96), 239 (4.40), 210 (4.13); IR (KBr): 3177 ν_OH_; 2845 ν_CH_; 1663 ν_C=O_; 1474 ν_C-H_.

*8-Hydroxyquinoline-7-carbaldehyde* (**2a’**) [4] ^1^H-NMR (DMSO–*d*_6_; 400.2 MHz) *δ* = 7.24 (d, ^3^*J*_H,H_ = 7.9 Hz, 1H, aromatic), 7.57 (dd, ^3^*J*_H,H_ = 8.1 Hz, ^4^*J*_H,H_ = 4.3 Hz, 1H, aromatic), 7.99 (d, ^3^*J*_H,H_ = 8.0 Hz, 1H, aromatic), 8.78 (dd, ^3^*J*_H,H_ = 4.4 Hz, ^4^*J*_H,H_ = 1.5 Hz, 1H, aromatic), 9.07 (dd, ^3^*J*_H,H_ = 8.0 Hz, ^4^*J*_H,H_ = 1.6 Hz, 1H, aromatic), 10.41 (s, 1H, HC=O); GC-MS: t_r_ = 6.360 min, (EI) *m*/*z* (rel. int.) M^+^ = 173 (12%); (M − CO + H)^+^ = 146 (100%).

*5-Chloro-8-hydroxyquinoline-7-carbaldehyde* (**2b**) yellow 0.5 g (2.5 mmol, 7.2%) [26]; m.p. = 170.0–170.6 °C; ^1^H-NMR (CDCl_3_; 500.18 MHz) *δ* = 7.71 (dd, ^3^*J*_H,H_ = 8.5 Hz, ^4^*J*_H,H_ = 4.2 Hz, 1H, aromatic), 7.86 (s, 1H, aromatic), 8.56 (dd, ^3^*J*_H,H_ = 8.5 Hz, ^4^*J*_H,H_ = 1.5 Hz, 1H, aromatic), 8.97 (dd, ^3^*J*_H,H_ = 4.2 Hz, ^4^*J*_H,H_ = 1.5 Hz, 1H, aromatic), 10.39 (s, 1H, HC=O); ^1^H-NMR (DMSO–*d*_6_; 500.18 MHz) *δ* = 7.74 (s, 1H, aromatic), 7.88 (dd, ^3^*J*_H,H_ = 8.5 Hz, ^4^*J*_H,H_ = 4.2 Hz, 1H, aromatic), 8.51 (dd, ^3^*J*_H,H_ = 8.5 Hz, ^4^*J*_H,H_ = 1.4 Hz, 1H, aromatic), 9.05 (dd, ^3^*J*_H,H_ = 4.2 Hz, ^4^*J*_H,H_ = 1.4 Hz, 1H, aromatic), 10.49 (s, 1H, HC=O); ^13^C{^1^H}-NMR (DMSO–*d*_6_; 125.78 MHz) *δ* = 118.7, 119.8, 122.7, 125.7, 129.3, 133.1, 140.2, 149.9, 158.4, 188.0; ^13^C{^1^H}-NMR (CDCl_3_; 125.78 MHz) *δ* = 117.8, 121.8, 124.7, 125.4, 130.2, 133.7, 139.9, 149.9, 157.6, 190.6; GC-MS: t_r_ = 6.917 min.; (EI) *m*/*z* (rel. int.) M^+^ = 207 (15%); (M − CO)^+^ = 179 (100%); UV-Vis (metanol; λ [nm] (logε)): 429 (2.86), 350 (3.27),286 (3.65), 267 (4.03), 248 (3.83), 207 (4.09); IR (KBr): 3344 ν_OH_; 2859 ν_CH_; 1667 ν_C=O_; 1425 ν_C-H_.

*5-Methyl-8-hydroxyquinoline-7-carbaldehyde* (**2c**) greenish 0.5 g; (2.8 mmol, 8.0%) [27]; m.p. = 172.7–173.5 °C; ^1^H-NMR (CDCl_3_; 400.2 MHz) *δ* = 2.56 (s, 3H, CH_3_), 7.53 (s, 1H, aromatic), 7.58 (dd, ^3^*J*_H,H_ = 8.2 Hz, ^4^*J*_H,H_ = 3.5 Hz, 1H, aromatic), 8.26 (d, ^3^*J*_H,H_ = 8.3 Hz, 1H, aromatic), 8.90 (d, ^3^*J*_H,H_ = 2.7 Hz, 1H, aromatic), 10.36 (s, 1H, HC=O); ^13^C{^1^H}-NMR (CDCl_3_; 100.6 MHz) *δ* = 17.9, 117.2, 124.3, 124.7, 124.9, 131.8, 133.0, 139.5, 148.9, 157.4, 192.3; GC-MS: t_r_ = 7.186 min.; (EI) *m*/*z* (rel. int.) M^+^ = 187 (19%); (M − CO)^+^ = 159 (100%); UV-Vis (metanol; λ [nm] (logε)): 452 (2.88), 426 (3.04), 358 (3.51), 291 (3.88), 270 (4.37), 246 (4.05), 207 (4.35); IR (KBr): 3063 ν_OH_; 2852 ν_CH_; 1686 ν_C=O_; 1426 ν_C-H_.

### 3.6. Synthesis of 7-Bromo-8-hydroxy-2-methylquinoline-5-carbaldehyde through a Carbene Insertion Reaction

Potassium hydroxide (40.0 g; 714.3 mmol) in water (15 mL) was added into the solution of **1a** (5.6 g; 18.0 mmol) in ethanol (20 mL). The resulting solution was irradiated (75 W) and stirred under reflux. Next, chloroform (30 mL, 372.0 mmol) was slowly added dropwise over an hour. The resulting red mixture was refluxed for another 16 h, and then cooled down to room temperature. Subsequently the obtained suspension was acidified by an aqueous solution of hydrochloric acid (1%) to pH ca. 7, and then volatiles were evaporated under reduced pressure. The resulting solid was dried over P_4_O_10_ and extracted at Soxhlet apparatus with chloroform. From the resulting solution the volatiles were evaporated under reduced pressure, and finally the crude product was purified on a silica gel chromatography with CH_3_Cl/MeOH (3:1) as eluent, and purified by crystallization from CH_3_Cl/hexane to yield precipitates as follows:

*7-Bromo-8-hydroxy-2-methylquinoline-5-carbaldehyde* (**2d**) < 1% [26]; ^1^H-NMR (DMSO–*d*_6_; 400.2 MHz) *δ* = 2.77 (s, 3H, CH_3_), 7.73 (d, ^3^*J*_H,H_ = 8.7 Hz, 1H, aromatic), 8.32 (s, 1H, aromatic), 9.45 (d, ^3^*J*_H,H_ = 8.7 Hz, 1H, aromatic), 10.05 (s, 1H, HC=O); GC-MS: t_r_ = 7.617 min.; (EI) *m*/*z* (rel. int.) M^+^ = 267 (15%); (M − CO + H)^+^ = 238 (20%).

### 3.7. General Procedures for Synthesis of Selected Quinoline-5-carbaldehydes Based on Vilsmeier-Haack Protocol

To the solution of dry chloroform (6.5 mL) and dry DMF (0.8 mL, 32.0 mmol) POCl_3_ (3.2 mL, 4.0 mmol) was added at 0 °C and the mixture was stirred for an hour. Next **1b**, **1e**, **1g** or **1i** (8.0 mmol), respectively was added, and the resulting reaction mixture was brought to a gentle reflux for 16 h. The reaction was quenched by the addition of crushed ice and was neutralized by aqueous solution of Na_2_CO_3_ (10%) to pH 6–7 and then the layers were separated. The aqueous layer was extracted by chloroform (3 × 30 mL), collected, and was dried over anhydrous MgSO_4_. After filtration, the solvent was evaporated on a rotary evaporator and the obtained residue was purified by column chromatography on silica gel with CH_3_Cl/THF/hexane (2:1:1) as eluent.

*6-(Dimethylamino)-2-methylquinoline-5-carbaldehyde* (**2e**) yellow 0.7 g (3.1 mmol, 38.6%); m.p. = 75.2–75.8 °C; ^1^H-NMR (DMSO–*d*_6_; 400.2 MHz) *δ* = 2.58 (s, 3H, CH_3_), 3.11 (s, 6H, 2NCH_3_), 7.43 (d, ^3^*J*_H,H_ = 8.8 Hz, 1H, aromatic), 7.64 (d, ^3^*J*_H,H_ = 9.4 Hz, 1H, aromatic), 7.98 (d, ^3^*J*_H,H_ = 9.4 Hz, 1H, aromatic), 9.18 (d, ^3^*J*_H,H_ = 8.8 Hz, 1H, aromatic), 10.20 (s, 1H, HC=O); ^13^C{^1^H}-NMR (DMSO–*d*_6_; 100.6 MHz) *δ* = 24.2, 45.7, 114.8, 121.9, 124.1, 125.2, 131.4, 135.2, 142.4, 155.6, 157.1, 190.4; GC-MS: t_r_ = 7.683 min, (EI) *m*/*z* (rel. int.) M^+^ = 214 (85%); (M − HCO)^+^ = 185 (55%); UV-Vis (methanol; λ [nm] (logε)): 416 (3.55), 365 (3.12), 304 (3.60), 289 (3.68), 260 (4.35), 214 (4.21); IR (KBr): 3386 ν_OH_; 2878 ν_CH_; 1671 ν_C=O_; 1498 ν_C-H_.

*6-(Dimethylamino)quinoline-5-carbaldehyde* (**2f**) yellow 1.2 g (5.9 mmol, 73.8%); m.p. = 56.1–56.8 °C; ^1^H-NMR (DMSO–*d*_6_; 400.2 MHz) *δ* = 3.16 (s, 6H, 2NCH_3_), 7.54 (dd, ^3^*J*_H,H_ = 8.7 Hz, ^4^*J*_H,H_ = 4.2 Hz, 1H, aromatic), 7.70 (d, ^3^*J*_H,H_ = 9.5 Hz, 1H, aromatic), 8.05 (d, ^3^*J*_H,H_ = 9.4 Hz, 1H, aromatic), 8.69 (dd, ^3^*J*_H,H_ = 4.2 Hz, ^4^*J*_H,H_ = 1.6 Hz, 1H, aromatic), 9.30 (dd, ^3^*J*_H,H_ = 8.7 Hz, ^4^*J*_H,H_ = 1.5 Hz, 1H, aromatic), 10.19 (s, 1H, HC=O); ^13^C{^1^H}-NMR (DMSO–*d*_6_; 125.8 MHz) *δ* = 45.5, 113.4, 122.7, 123.6, 127.5, 132.0, 134.7, 141.6, 146.6, 157.5, 190.0; GC-MS: t_r_ = 7.259 min, (EI) *m*/*z* (rel. int.) M^+^ = 200 (76%); (M − HCO)^+^ = 171 (20%); UV-Vis (methanol; λ [nm] (logε)): 423 (3.61), 373 (3.18), 307 (3.60), 268 (4.33), 222 (4.17), 204 (3.96); IR (KBr): 3421 ν_OH_; 2895 ν_CH_; 1636 ν_C=O_; 1458 ν_C-H_.

*8-(Dimethylamino)quinoline-5-carbaldehyde* (**2i**) yellow 0.01 g (0.05 mmol, 0.6%); m.p. = 100.8–101.0 °C; ^1^H-NMR (CDCl_3_; 400.2 MHz) *δ* = 3.36 (s, 6H, 2NCH_3_), 6.97 (d, ^3^*J*_H,H_ = 8.2 Hz, 1H, aromatic), 7.53 (dd, ^3^*J*_H,H_ = 8.6 Hz, ^4^*J*_H,H_ = 4.1 Hz, 1H, aromatic), 7.84 (d, ^3^*J*_H,H_ = 8.3 Hz, 1H, aromatic), 8.87 (dd, ^3^*J*_H,H_ = 4.0 Hz, ^4^*J*_H,H_ = 1.7 Hz, 1H, aromatic), 9.72 (dd, ^3^*J*_H,H_ = 8.6 Hz, ^4^*J*_H,H_ = 1.7 Hz, 1H, aromatic), 10.06 (s, 1H, HC=O); ^13^C{^1^H}-NMR (CDCl_3_; 125.8 MHz) *δ* = 44.1, 111.2, 122.2, 123.3, 128.3, 133.9, 139.5, 141.0, 146.9, 155.0, 191.3; GC-MS: t_r_ = 7.156 min, (EI) *m*/*z* (rel. int.) M^+^ = 200.1 (24%), (M − Me)^+^ = 185.1 (100%), (M − HCO)^+^ = 171.1 (38%); UV-Vis (methanol; λ [nm] (logε)): 390 (4.07), 289 (3.91), 263 (4.20), 234 (3.99), 206 (4.36); IR (KBr): 2840 ν_CH_; 1665 ν_C=O_; 1555; 1509 ν_C-H_; 1352; 1251; 1080; 760.

*8-(Dimethylamino)quinoline-5,7-dicarbaldehyde* (**2j**) yellow 0.006 g (0.002 mmol, 0.3%); m.p. = 100.0–100.1 °C; ^1^H-NMR (CDCl_3_; 400.2 MHz) *δ* = 3.61 (s, 6H, 2NCH_3_), 7.57 (dd, ^3^*J*_H,H_ = 8.6 Hz, ^4^*J*_H,H_ = 4.1 Hz, 1H, aromatic), 8.26 (s, 1H, aromatic), 8.88 (d, ^3^*J*_H,H_ = 4.1 Hz, ^4^*J*_H,H_ = 1.7 Hz, 1H, aromatic), 9.70 (dd, ^3^*J*_H,H_ = 8.6 Hz, ^4^*J*_H,H_ = 1.7 Hz, 1H, aromatic), 10.06 (s, 1H, HC=O), 10.17 (s, 1H, HC=O); ^13^C{^1^H}-NMR (CDCl_3_; 125.8 MHz) *δ* = 48.3, 122.1, 122.6, 124.7, 130.2, 134.0, 141.9, 144.4, 147.5, 157.0, 188.5, 191.3; GC-MS: t_r_ = 7.952 min, (EI) *m*/*z* (rel. int.) M^+^ = 227.9 (100%), (M − Me)^+^ = 213.0 (3%), (M + 2H − CO)^+^ = 202 (10%); LCMS-IT-TOF: *m*/*z* (rel. int.) (M + H)^+^ = 229 (100%), (M + H − CO)^+^ = 201 (100%); HRMS (IT-TOF): *m*/*z* Calcd for C_13_H_13_N_2_O_2_ (M + H)^+^ = 229.0977, Found 229.0970; UV-Vis (methanol; λ [nm] (logε)): 400 (4.33), 355 (4.04), 287 (4.38), 234 (4.33), 210 (4.33); IR (KBr): 2872 ν_CH_; 1678 ν_C=O_; 1661 ν_C=O_; 1515 ν_C-H_; 1381, 1265, 1103, 765.

*(Z)-8-Hydroxy-2-(2-hydroxyvinyl)quinoline-5-carbaldehyde* (**2l**) yellow 0.6 g (2.6 mmol, 32.1%); m.p. = 153.1–153.8 °C; ^1^H-NMR (DMSO–*d*_6_; 400.2 MHz) *δ* = 7.28 (dd, ^3^*J*_H,H_ = 5.7 Hz, ^4^*J*_H,H_ = 3.2 Hz, 1H, aromatic), 7.45–7.48 (m, 2H, aromatic), 8.58 (d, ^3^*J*_H,H_ = 9.3 Hz, 1H, aromatic), 8.86 (d, ^3^*J*_H,H_ = 9.2 Hz, 1H, aromatic), 9.43 (s, 2H, HC=O), 11.42 (s, 1H, OH), 16.14 (s, 1H, OH); ^13^C{^1^H}-NMR (DMSO–*d*_6_; 125.8 MHz) *δ* = 106.4, 115.1, 118.0, 118.1, 125.4, 125.9, 126.9, 142.1, 146.4, 150.7, 189.6, 191.8; LCMS-IT-TOF: *m*/*z* (rel. int.) (M + H)^+^ = 216 (100%); HRMS (IT-TOF): *m*/*z* Calcd for C_12_H_10_NO_3_ (M + H)^+^ = 216.0660, Found 216.0665; UV-Vis (methanol; λ [nm] (logε)): 402 (3.91), 382 (3.99), 297 (4.28), 262 (4.07), 241 (4.21), 214 (4.39); IR (KBr): 3102 ν_OH_; 2906 ν_CH_; 1594 ν_C=O_; 1353 ν_C-H_.

### 3.8. General Procedures for the Synthesis of Selected Quinolinecarbaldehydes Based on the Duff Protocol

These were based on a procedure described in the literature [28]. To a solution of **1b**, **1d**, **1e**, **1f**, **1h** or **1j** (5.0 mmol) in a minimum amount of TFA (7–8 mL) hexamethylenetetramine (1.4 g, 10.0 mmol) was gently added under an argon atmosphere. The solution was stirred at 70 °C for 70 h and then at 100 °C for another 4 h. Subsequently, the obtained suspension was acidified by an aqueous solution of hydrochloric acid (10%, ~10 mL) and the reaction mixture was kept at 100 °C for 1 h. The whole suspension was cooled down to r.t. Next, the obtained reaction mixture was alkalified by aqueous solution of NaOH (10%), and the resulting precipitate was collected in a Buchner funnel, followed by washing with water (3 × 50 mL) and dried to afford a solid. Next, the crude product was purified by chromatography to yield **2c** precipitates as follows, or the crude product was extracted with CH_2_Cl_2_ at Soxhlet apparatus to yield **2h** solid as follows:

*5-Chloro-8-hydroxyquinoline-7-carbaldehyde* (**2b**) 0.7 g (3.5 mmol, 70%).

*5-Methyl-8-hydroxyquinoline-7-carbaldehyde* (**2c**) 0.7 g (3.7 mmol; 75.0%).

*6-Hydroxyquinoline-5-carbaldehyde* (**2g**) beige 0.6 g (3.5 mmol, 28.1%) [29]; m.p. = 138.6–138.9 °C; ^1^H-NMR (CDCl_3_; 400.2 MHz) *δ* = 7.39 (d, ^3^*J*_H,H_ = 9.3 Hz, 1H, aromatic), 7.53 (dd, ^3^*J*_H,H_ = 8.6 Hz, ^4^*J*_H,H_ = 4.2 Hz, 1H, aromatic), 8.26 (d, ^3^*J*_H,H_ = 9.3 Hz, 1H, aromatic), 8.67 (d, ^3^*J*_H,H_ = 8.6 Hz, 1H, aromatic), 8.85 (d, ^3^*J*_H,H_ = 3.1 Hz, 1H, aromatic), 10.76 (s, 1H, HC=O), 13.06 (s, 1H, OH); ^13^C{^1^H}-NMR (CDCl_3_; 100.6 MHz) *δ* = 110.7, 123.1, 123.5, 127.0, 128.2, 140.7, 143.4, 148.7, 164.9, 192.4; GC-MS: t_r_ = 6.168 min, (EI) *m*/*z* (rel. int.) M^+^ = 173 (100%); (M − HCO)^+^ = 144 (20%); UV-Vis (methanol; λ [nm] (logε)): 400 (2.46), 346 (3.16), 300 (3.43), 290 (3.37), 226 (4.04), 203 (0.98); IR (KBr): 3050 ν_OH_; 2733 ν_CH_; 1632 ν_C=O_; 1480 ν_C-H_.

*8-Hydroxy-2-methylquinoline-5,7-dicarbaldehyde* (**2h**) red 0.2 g (0.7 mmol, 14.9%); m.p. > 360 °C; ^1^H-NMR (DMSO–*d*_6_; 400.2 MHz) *δ* = 2.85 (s, 3H, CH_3_), 7.92 (d, ^3^*J*_H,H_ = 8.7 Hz, 1H, aromatic), 8.29 (s, 1H, aromatic), 9.71 (d, ^3^*J*_H,H_ = 8.7 Hz, 1H, aromatic), 9.95 (s, 1H, HC=O), 10.42 (s, 1H, HC=O); ^13^C{^1^H}-NMR (DMSO–*d*_6_; 125.8 MHz) *δ* = 21.6, 115.6, 119.5, 128.0, 128.6, 137.8, 138.5, 139.0, 155.7, 167.2, 188.2, 191.5; LCMS-IT-TOF: *m*/*z* (rel. int.) (M − H)^−^ = 214 (100%), M^−^ = 215 (10%); (M − HCO)^−^ = 186 (10%); (M − 2HCO)^−^ = 157 (<1%); HRMS (IT-TOF): *m*/*z* Calcd for C_12_H_8_NO_3_ (M − H)^−^ = 214.0504, Found 214.0496; UV-Vis (methanol; λ [nm] (logε)): 359 (3.65), 282 (3.91), 237 (3.59); IR (KBr): 3423 ν_OH_; 2965 ν_CH_; 2847 ν_CH_; 2835 ν_CH_; 1657 ν_C=O_; 1458 ν_C-H_; CCDC 1890715.

*10-hydroxybenzo[h]quinoline-7,9-dicarbaldehyde* (**2k**) red 0.9 g (3.5 mmol, 70.6%); m.p._dec._ > 360 °C; ^1^H-NMR (DMSO–*d*_6_/KOD/D_2_O; 400.2 MHz) *δ* = 7.51 (dd, ^3^*J*_H,H_ = 8.0 Hz, ^4^*J*_H,H_ = 4.2 Hz, 1H, aromatic), 7.96 (d, ^3^*J*_H,H_ = 9.0 Hz, 1H, aromatic), 8.10 (s, 1H, aromatic), 8.26 (dd, ^3^*J*_H,H_ = 8.1 Hz, ^4^*J*_H,H_ = 2.0 Hz, 1H, aromatic), 8.89 (dd, ^3^*J*_H,H_ = 4.2 Hz, ^4^*J*_H,H_ = 2.0 Hz, 1H, aromatic), 9.20 (d, ^3^*J*_H,H_ = 9.0 Hz, 1H, aromatic), 9.65 (s, 1H, HC=O), 10.08 (s, 1H, HC=O); ^13^C{^1^H}-NMR (DMSO–*d*_6_/KOD/D_2_O; 125.8 MHz) *δ* = 115.1, 122.2, 123.8, 124.8, 126.1, 126.9, 132.3, 137.4, 140.0, 144.6, 148.1, 151.1, 182.0, 192.6, 192.7; HRMS (ESI): *m*/*z* Calcd for C_15_H_9_NO_3_ M^−^ = 251.05826, Found 251.07750; UV-Vis (methanol; λ [nm] (logε)): 453 (2.41), 405 (3.22), 364 (3.38), 329 (3.46), 315 (3.48), 274 (3.96), 260 (4.03), 241 (4.02), 223 (4.01), 211 (4.04); IR (KBr): 3424 ν_OH_; 3062 ν_CH_; 2877 ν_CH_; 1673 ν_C=O_; 1483 ν_C-H_.

*10-Hydroxybenzo[h]quinoline-7-carbaldehyde* [30] < 1%.

*10-Hydroxybenzo[h]quinoline-9-carbaldehyde* [31] < 1%.

### 3.9. General Procedure the for Synthesis of Selected Schiff Base Derivatives of 2,6-Diisopropylbenzenamine

Compounds **2a**, **2c**, **2e** or **2f** (2.0 mmol) and 2,6-diisopropylaniline (0.5 g; 0.565 mL; 3.0 mmol) were dissolved in dry chloroform (70 mL) and then the resulting reaction mixture was brought to a gentle reflux for 40 h. The reaction mixture was connected with a Soxhlet apparatus in which MgSO_4_ were placed as dehydrating agent. Next the solvent was evaporated under reduced pressure and the obtained red residue was purified by chromatography and crystallization from acetonitrile to yield precipitates as follows:

*5-[(E)-{[2,6-Di(propan-2-yl)phenyl]imino}methyl]quinolin-8-ol* (**3a**) orange 0.5 g (1.4 mmol, 71.1%); m.p. = 126.1–126.9 °C; ^1^H-NMR (CDCl_3_; 400.2 MHz) *δ* = 1.20 (d, ^3^*J*_H,H_ = 6.9 Hz, 12H, 4CH_3_), 3.06 (hept, ^3^*J*_H,H_ = 6.9 Hz, 2H, 2C*H*), 7.10–7.24 (m, 3H, aromatic), 7.35 (d, ^3^*J*_H,H_ = 8.0 Hz, 1H, aromatic), 7.66 (dd, ^3^*J*_H,H_ = 8.7 Hz, ^4^*J*_H,H_ = 4.3 Hz, 1H, aromatic), 7.84 (d, ^3^*J*_H,H_ = 8.0 Hz, 1H, aromatic), 8.51 (s, 1H, HC=N), 8.91 (dd, ^3^*J*_H,H_ = 4.3 Hz, ^4^*J*_H,H_ = 1.5 Hz, 1H, aromatic), 10.04 (d, ^3^*J*_H,H_ = 8.7 Hz, 1H, aromatic); ^13^C{^1^H}-NMR (CDCl_3_; 100.6 MHz) *δ* = 23.7, 28.3, 109.5, 122.7, 123.2, 123.7, 124.2, 127.3, 135.1, 135.6, 137.9, 138.3, 148.1, 149.9, 155.2, 162.7; LCMS-IT-TOF: *m*/*z* (rel. int.) (M + H)^+^ = 333 (100%); HRMS (IT-TOF): *m*/*z* Calcd for C_22_H_25_N_2_O (M + H)^+^ = 333.1961, Found 333.1965; UV-Vis (methanol; λ [nm] (logε)): 327 (3.88), 271 (3.94), 239 (4.38), 206 (4.43); IR (KBr): 3381 ν_OH_; 2858 ν_CH_; 2958 ν_OH_; 1613 ν_HC=N_. CCDC 1501807.

*5-[(E)-{[2,6-Di(propan-2-yl)phenyl]imino}methyl]-N,N,2-trimethylquinolin-6-amine* (**3b**) yellow 0.6 g (1.5 mmol, 74.3%); m.p. = 130.1–130.5 °C; ^1^H-NMR (CDCl_3_; 400.2 MHz) *δ* = 1.20 (d, ^3^*J*_H,H_ = 6.9 Hz, 12H, 4CH_3_), 2.77 (s, 3H, CH_3_), 2.90 (s, 6H, 2NCH_3_), 3.11 (hept, ^3^*J*_H,H_ = 6.9 Hz, 2H, 2CH), 7.14 (dd, ^3^*J*_H,H_ = 8.6 Hz, ^3^*J*_H,H_ = 6.6 Hz, 1H, aromatic), 7.19–7.23 (m, 2H, aromatic), 7.38 (d, ^3^*J*_H,H_ = 8.9 Hz, 1H, aromatic), 7.64 (d, ^3^*J*_H,H_ = 9.2, 1H, aromatic), 8.18 (d, ^3^*J*_H,H_ = 9.2 Hz, 1H, aromatic), 8.80 (s, 1H, HC=N), 10.00 (d, ^3^*J*_H,H_ = 8.9, 1H, aromatic); ^13^C{^1^H}-NMR (CDCl_3_; 100.6 MHz) *δ* = 23.9, 24.7, 28.1, 46.2, 121.3, 122.7, 123.3, 123.7, 124.2, 126.1, 132.5, 135.4, 137.9, 144.1, 150.17, 155.1, 157.1, 162.5; LCMS-IT-TOF: *m*/*z* (rel. int.) (M + H)^+^ = 374 (100%); HRMS (IT-TOF): *m*/*z* Calcd for C_25_H_32_N_3_ (M + H)^+^ = 374.2590, Found 374.2588; UV-Vis (methanol; λ [nm] (logε)): 377 (3.87), 306 (4.06), 260 (4.59), 214 (4.71); IR (KBr): 2959 ν_CH_; 1629 ν_HC=N_. CCDC 1501808.

*5-[(E)-{[2,6-Di(propan-2-yl)phenyl]imino}methyl]-N,N-dimethylquinolin-6-amine* (**3c**) dark yellow 0.6 g (1.6 mmol, 80.3%); m.p. = 116.9–117.2 °C; ^1^H-NMR (CDCl_3_; 400.2 MHz) *δ* = 1.25 (d, ^3^*J*_H,H_ = 6.9 Hz, 12H, 4CH_3_), 2.97 (s, 6H, 2NCH_3_), 3.14 (hept, ^3^*J*_H,H_ = 6.9 Hz, 2H, 2CH), 7.16–7.32 (m, 3H, aromatic), 7.53 (dd, ^3^*J*_H,H_ = 8.8 Hz, ^4^*J*_H,H_ = 4.1 Hz, 1H, aromatic), 7.72 (d, ^3^*J*_H,H_ = 9.2 Hz, 1H, aromatic), 8.27 (d, ^3^*J*_H,H_ = 9.2 Hz, 1H, aromatic), 8.84 (s, 1H, HC=N), 8.89 (dd, ^3^*J*_H,H_ = 4.0 Hz, ^4^*J*_H,H_ = 1.4 Hz, 1H, aromatic), 10.15 (d, ^3^*J*_H,H_ = 8.7 Hz, 1H, aromatic); ^13^C{^1^H}-NMR (CDCl_3_; 100.6 MHz) *δ* = 23.9, 28.1, 46.2, 120.8, 122.6, 122.7, 123.2, 124.2, 128.0, 133.5, 134.9, 137.9, 144.8, 148.4, 150.1, 155.7, 162.3; LCMS-IT-TOF: *m*/*z* (rel. int.) (M + H)^+^ = 360 (100%); HRMS (IT-TOF): *m*/*z* Calcd for C_24_H_30_N_3_ (M + H)^+^ = 360.2434, Found 360.2432; UV-Vis (methanol; λ [nm] (logε)): 379 (3.62), 305 (3.80), 263 (4.29), 213 (4.42); IR (KBr): 2959 ν_CH_; 1619 ν_HC=N_. CCDC 1829344.

*(E)-7-(((2,6-Diisopropylphenyl)imino)methyl)-5-methylquinolin-8-ol* (**3d**) yellow 0.5 g (1.6 mmol, 78.0%); m.p. = 175.1–176.1 °C; ^1^H-NMR (CDCl_3_; 500.18 MHz) *δ* = 1.25 (d, ^3^*J*_H,H_ = 6.9 Hz, 12H, 4CH_3_), 2.63 (d, ^4^*J*_H,H_ = 0.9 Hz, 3H, CH_3_), 3.09 (hept, ^3^*J*_H,H_ = 6.8 Hz, 2H, 2CH), 7.23–7.24 (m, 4H, aromatic), 7.58 (dd, ^3^*J*_H,H_ = 8.5 Hz, ^4^*J*_H,H_ = 4.2 Hz, 1H, aromatic), 8.28 (dd, ^3^*J*_H,H_ = 8.5 Hz, ^4^*J*_H,H_ = 1.6 Hz, 1H, aromatic), 8.38 (s, 1H, HC=N), 9.03 (dd, ^3^*J*_H,H_ = 4.2 Hz, ^4^*J*_H,H_ = 1.6 Hz, 1H, aromatic), 14.46 (s, 1H, OH); ^13^C{^1^H}-NMR (CDCl_3_; 125.78 MHz) *δ* = 18.1, 23.7, 28.3, 113.9, 123.1, 123.3, 123.5, 126.1, 127.5, 130.9, 132.5, 139.7, 141.3, 144.5, 149.1, 161.2, 165.3; LCMS-IT-TOF: *m*/*z* (rel. int.) (M − H)^−^ = 345 (100%); HRMS (IT-TOF): *m*/*z* Calcd for C_23_H_25_N_2_O (M − H)^−^ = 345.1967, Found 345.1969; UV-Vis (methanol; λ [nm] (logε)): 446 (2.98), 361 (3.17), 277 (3.93), 226 (3.71), 208 (4.02); IR (KBr): 3062 ν_OH_; 2962 ν_CH_; 1617 ν_HC=N_. CCDC 1829345.

### 3.10. Crystallization

Crystals suitable for X-ray analysis were obtained from a hot acetonitrile solution for **3a**, **3b**, **3c** and **3d**, and from a hot CHCl_3_ solution for **2h**.

## 4. Conclusions

The research has been focused on the synthesis of quinolinecarbaldehydes **2** and Schiff bases **3** as their derivatives. The presented synthesis protocols allowed the synthesis of the target compounds more efficiently with yields up to 75% for molecule **2c** and 80.3% for compound **3c**. The structures of the obtained molecules were proved by a combination of various techniques, such as NMR, IR, GC-MS, MS, HRMS, UV-Vis and X-ray crystallography. The chemistry was mostly based on inexpensive and commercially available reagents. A variety of substituents (halogens Cl and Br and hydroxyl, methyl and NMe_2_ groups) were chosen in order to represent different electronic features. Formylation reactions of electron-rich aromatics in our studies mainly led to quinoline-5-carbaldehyde structures with newly formed carbonyl groups at the C5 position. In the case of a C5 position blocked by bromine or chlorine atoms or methyl groups, formylation reactions produced quinoline-7-carbaldehydes with the carbonyl group in C7 position. We presented a very simple, chromatography-free procedure for the double formylation of 2-methylquinolin-8-ol and benzo[*h*]quinolin-10-ol using very convenient Duff reaction protocols. For the first time we showed a carbene insertion reaction into C-Br bonds to produce 7-bromo-8-hydroxyquinoline-5-carbaldehyde by applying the Reimer-Tiemann method. The electrochemical properties of compounds **2a**, **2e** and **2f** were investigated. Oxidation and reduction potentials of these compounds in acetonitrile showed the influence of the various functional groups in the structure.

## Figures and Tables

**Table 1:** Crystal data and structure refinement details of compounds **2h**, **3a**, **3b**, **3c** and **3d**.

	2h	3a	3b	3c	3d
Empirical formula	C_12_H_9_NO_3_,CHCl_3_	3(C_22_H_24_N_2_O)·CH_3_CN	C_25_H_31_N_3_	C_24_H_29_N_3_	C_23_H_26_N_2_O
Temperature (K)	100(1)	100(1)	100(1)	293(1)	80(1)
Wavelength (Å)		0.71073 (Mo Kα)			
Crystal system	Orthorhombic	Trigonal	Orthorhombic	Monoclinic	Monoclinic
Space group	Pnma	*P*3_1_c	*P*bca	*P*2_1_/c	*P*2_1_/c
Unit cell dimensions					
a (Å)	26.1040(14)	29.4970(4)	18.8459(8)	9.0651(3)	24.0519(9)
b (Å)	6.4668(4)	29.4970(4)	11.0427(3)	11.2850(3)	16.2882(7)
c (Å)	8.2056(4)	12.0258(3)	20.8067(6)	20.8767(7)	9.7079(3)
α (o)	90	90	90	90	90
β (o)	90	90	90	93.873(3)	99.189(3)
γ (o)	90	120	90	90	90
Volume (Å^3^)	1385.18(13)	9061.5(3)	4330.1(3)	2130.80(12)	3754.4(2)
Z	4	6	8	4	8
Calculated density (Mg/m^3^)	1.604	1.142	1.146	1.121	1.226
Absorption coefficient (mm^−1^)	0.666	0.070	0.068	0.066	0.075
F (000)	680	3336	1616	776	1488
Crystal dimensions (mm)	0.12 × 0.10 × 0.02	0.18 × 0.13 × 0.03	0.25 × 0.02 × 0.02	0.56 × 0.22 × 0.09	0.30 × 0.05 × 0.02
*θ* range for data collection (^o^)	2.932 to 26.371	2.875 to 24.710	3.452 to 26.370	3.401 to 26.367	3.033 to 26.371
Index ranges	−32 ≤ *h* ≤ 32; −6 ≤ *k* ≤ 8; −10 ≤ *l* ≤ 10	−34 ≤ *h* ≤ 34; −34 ≤ *k* ≤ 31; −14 ≤ *l* ≤ 14	−21 ≤ *h* ≤ 23; −13 ≤ *k* ≤ 9; −26 ≤ *l* ≤ 26	−11 ≤ *h* ≤ 11; −14 ≤ *k* ≤ 9; −26 ≤ *l* ≤ 26	−30 ≤ *h* ≤ 30; −20 ≤*k* ≤ 20; −9 ≤ *l* ≤ 12
Reflections collected	13863	69751	25986	25693	30149
Independent reflections	1543 [R(int) = 0.0162]	10284 [R(int) = 0.0713]	4420 [R(int) = 0.0680]	4343 [R(int) = 0.0509]	7669 [R(int) = 0.0599]
Data/restraints/parameters	1543/0/120	10284/1/721	4420/0/260	4343/0/251	7669/0/493
Goodness-of-fit on F^2^	1.223	1.070	1.072	1.026	1.032
Final R indices (I > 2σ(I))	R_1_ = 0.0547; wR_2_ = 0.1379	R_1_ = 0.0581; wR_2_ = 0.1287	R_1_ = 0.0488; wR_2_ = 0.1082	R_1_ = 0.0494; wR_2_ = 0.1180	R_1_ = 0.0521; wR_2_ = 0.1228
R indices (all data)	R_1_ = 0.0556; wR_2_ = 0.1385	R_1_ = 0.0811; wR_2_ = 0.1436	R_1_ = 0.0776; wR_2_ = 0.1262	R_1_ = 0.0763; wR_2_ = 0.1329	R_1_ = 0.0884; wR_2_ = 0.1499
Largest diff. Peak and hole	0.696 and −0.377	0.159 and −0.257	0.203 and −0.191	0.158 and −0.154	0.325 and −0.297
CCDC-Number	1890715	1501807	1501808	1829344	1829345

**Table 2:** Selected bond lengths and angles of compounds **2h**, **3a**, **3b**, **3c** and **3d** (Å and ^o^).

	Bond Lengths (Å)				
	2h	3a	3b	3c	3d
C(10)–N(2)	-	1.269(6)	1.2741	1.265(2)	1.2816
C(33)–N(4)	-	1.421(6)	1.417(2)	1.426(2)	1.4263
C(5)–C(10)	-	1.459(7)	1.465(2)	1.4631	1.4488
C(5)–C(11)	1.448	-	-	-	-
C(7)–C(12)	1.452	-	-	-	-
C(8)–O(1)	1.243	1.350(5)	-	-	1.341
C(6)–N(3)	-	-	1.415(2)	1.403(2)	-
	Angles (^o^)				
C(10)–N(2)–C(11)		116.8(4)	120.5(1)	117.5(1)	121.18
C(5)–C(10)–N(2)		127.2(5)	124.0(1)	126.1(1)	121.52

## Supplementary Materials

The following are available online, CCDC 1890715, 1501807, 1501808, 1829344 and 1829345 for **2h**, **3a**, **3b**, **3c** and **3d**, respectively contains the supplementary crystallographic data for the compounds. These data can be obtained free of charge from http://www.ccdc.cam.ac.uk/conts/retrieving.html, or from the Cambridge Crystallographic Data Centre, 12 Union Road, Cambridge CB2 1EZ, UK; fax: (+44) 1223-336-033; or e-mail: deposit@ccdc.cam.ac.uk. Calculations have been carried out in Wroclaw Centre for Networking and Supercomputing (http://www.wcss.wroc.pl).
